# Ingested Ketone Ester Leads to a Rapid Rise of Acetyl-CoA and Competes with Glucose Metabolism in the Brain of Non-Fasted Mice

**DOI:** 10.3390/ijms22020524

**Published:** 2021-01-07

**Authors:** Laurent Suissa, Pavel Kotchetkov, Jean-Marie Guigonis, Emilie Doche, Ophélie Osman, Thierry Pourcher, Sabine Lindenthal

**Affiliations:** 1 Laboratory Transporter in Imaging and Radiotherapy in Oncology (TIRO), Direction de la Recherche Fondamentale (DRF), Institut des Sciences du Vivant Fréderic Joliot, Commissariat à l’Energie Atomique et aux Énergies Alternatives (CEA), University Côte d’Azur, F-06107 Nice, France; laurent.suissa@ap-hm.fr (L.S.); kotchetkov.pavel@gmail.com (P.K.); Jean-Marie.GUIGONIS@univ-cotedazur.fr (J.-M.G.); Thierry.Pourcher@unice.fr (T.P.); 2 Stroke Unit, University Hospital, F-13005 Marseille, France; emilie.doche@ap-hm.fr (E.D.); ophelie.osman@ap-hm.fr (O.O.)

**Keywords:** ketosis, ketogenic diet, exogenous ketone bodies, ketone ester, β-hydroxybutyrate, cerebral energy metabolism

## Abstract

The role of ketone bodies in the cerebral energy homeostasis of neurological diseases has begun to attract recent attention particularly in acute neurological diseases. In ketogenic therapies, ketosis is achieved by either a ketogenic diet or by the administration of exogenous ketone bodies. The oral ingestion of the ketone ester (KE), (R)-3-hydroxybutyl (R)-3-hydroxybutyrate, is a new method to generate rapid and significant ketosis (i.e., above 6 mmol/L) in humans. KE is hydrolyzed into β-hydroxybutyrate (βHB) and its precursor 1,3-butanediol. Here, we investigate the effect of oral KE administration (3 mg KE/g of body weight) on brain metabolism of non-fasted mice using liquid chromatography in tandem with mass spectrometry. Ketosis (Cmax = 6.83 ± 0.19 mmol/L) was obtained at Tmax = 30 min after oral KE-gavage. We found that βHB uptake into the brain strongly correlated with the plasma βHB concentration and was preferentially distributed in the neocortex. We showed for the first time that oral KE led to an increase of acetyl-CoA and citric cycle intermediates in the brain of non-fasted mice. Furthermore, we found that the increased level of acetyl-CoA inhibited glycolysis by a feedback mechanism and thus competed with glucose under physiological conditions. The brain pharmacodynamics of this oral KE strongly suggest that this agent should be considered for acute neurological diseases.

## 1. Introduction

Ketone body metabolism is a survival trait that is conserved among higher organisms to prolong life during an energy deficit or a metabolic crisis. In certain circumstances (such as fasting or diabetes mellitus), ketone bodies produced by the liver for the most part from fatty acids, act mainly as respiratory fuels to power oxidative phosphorylation in various organs including the brain. The main benefit of ketone bodies is their ability to act as an alternative energy source to glucose for mitochondrial ATP production [1,2].

Recently, the contribution of ketone bodies to cerebral energy homeostasis has generated interest, particularly in chronic but also acute neurological diseases because mitochondrial dysfunction and altered glucose metabolism are thought to play an important role in the progression of these pathologies [3,4,5,6,7]. Since the 1980s, several pre-clinical studies have consistently shown the potential for ketogenic therapy in the acute neuroprotection of cerebral ischemic injuries in animal models [8,9,10,11,12,13]. In ketogenic therapies, ketosis is commonly achieved by either a ketogenic diet (carbohydrate privation) or by administration of exogenous ketone bodies (β-hydroxybutyrate, acetoacetate) or their precursors (1,3-butanediol). Described as pleiotropic, the underlying molecular mechanisms of the neuroprotective effects of ketogenic therapies are still uncertain but they are thought to be principally based on the more energy-efficient metabolism of ketone bodies compared to glucose [14]. To our knowledge, the translation of these results into clinical studies was not proposed due to the ketosis induction methods used in these animal models that are not suitable for use with humans. Applying a ketogenic diet is not suitable in the case of acute diseases because of the delayed onset of ketosis.

Ketone esters have been developed over the last decade and are now commercially available, which enables rapid and significant ketosis in humans. Oral ingestion of the KE, (R)-3-hydroxybutyl (R)-3-hydroxybutyrate, was confirmed by the Food and Drug Administration to be a safe method to increase the plasma ketone level in humans (GRAS substance: Generally Recognized as Safe). Increased blood d-β-hydroxybutyrate (d-βHB) concentration can be rapidly obtained with a simple ketone drink that bypasses any dietary restrictions and does not increase acid or salt loads when exogenous Na-βHB or βHB acid forms are used [15].

In this pre-clinical study, we describe the brain pharmacokinetic and the brain pharmacodynamic of the ketone ester (R)-3-hydroxybutyl (R)-3-hydroxybutyrate in KE-gavaged, non-fasted mice. Here we report, for the first time, that rapid and significant ketosis induced by a single oral KE intake led to a fast increase of the β-hydroxybutyrate and acetyl-CoA levels in the brain of these non-fasted mice. The animals also showed an increase in the brain C_4_OH-carnitine level that is a storage form of β-hydroxybutyrate, and in the levels of the citric cycle intermediates. We therefore concluded that the β-hydroxybutyrate was either stored as C_4_OH-carnitine or directly entered the citric acid cycle via the acetyl-CoA and was thus competing with glucose metabolism.

## 2. Results

### 2.1. Plasma Analysis of (R)-3-Hydroxybutyl (R)-3-Hydroxybutyrate-Gavaged Mice

**Figure 1:**
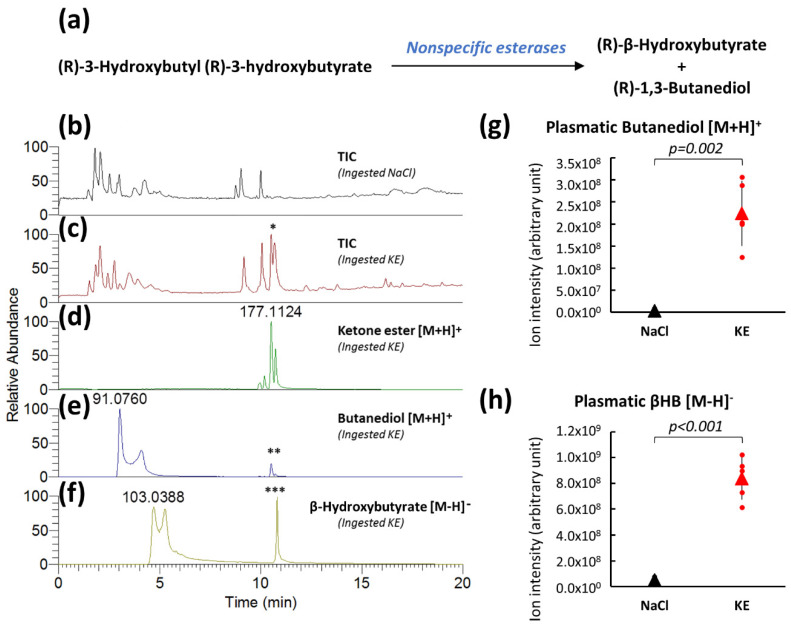
Plasma analyses after KE ingestion. Plasma β-hydroxybutyrate (βHB)and 1,3-butanediol levels (**a**). Total ion LC-MS chromatograms (TIC) acquired in positive spectrometry mode of plasma samples from 0.9% NaCl-gavaged control mice (**b**) from KE-gavaged mice (3 mg KE/g of body weight) (**c**) (* KE peak on the TIC chromatogram). Extracted ion chromatograms of KE (**d**) and its metabolites butanediol (**e**) and β-hydroxybutyrate (**f**) from plasma of KE-gavaged mice (in electrospray ionization source produced butanediol from KE (**) and β-hydroxybutyrate from KE (***). Levels of KE metabolites (butanediol [M+H]^+^ (**g**) and β-hydroxybutyrate [M−H]^−^ (**h**)) from the plasma of mice 30 min after NaCl or KE gavage (n = 5).

Aliphatic esters, such as (R)-3-hydroxybutyl (R)-3-hydroxybutyrate are hydrolyzed by nonspecific esterases into (R)-β-hydroxybutyrate and (R)-1,3-butanediol (Figure 1a) in the small intestine, the liver, and the blood [16]. Both resulting metabolites are absorbed into the portal circulation, with butanediol undergoing first-pass metabolism in the liver to form β-hydroxybutyrate [17,18], which is subsequently released into the circulating blood.

Plasma samples of non-fasted mice 30 min after the oral intake of 3 mg of KE ((R)-3-hydroxybutyl (R)-3-hydroxybutyrate)/g of body weight or of control solution (0.9% NaCl) were obtained and analyzed by LC-MS (Liquid Chromatography—Mass Spectrometry) to determine the levels of KE and its metabolites, butanediol and β-hydroxybutyrate. KE was found to be present in the plasma from non-fasted KE-gavaged mice by total LC-MS ionic chromatograms (TIC) acquired in positive spectrometry mode, with the appearance of a split peak at the retention times of 10.48 min that was not present in plasma samples from control mice (Figure 1b,c). Representative extracted ion chromatograms of the (R)-β-hydroxybutyl (R)-3-hydroxybutyrate ([M+H]^+^ ions at *m*/*z* 177.1121) from the plasma of KE-gavaged mice are shown in Figure 1d. A split peak was also found at the same retention time (i.e., 10.48 min). Degradation of KE resulted in butanediol ([M+H]^+^ ions at *m*/*z* 91.0754) and β-hydroxybutyrate ([M−H]^−^ ions at *m*/*z* 103.0401). Representative extracted ion chromatograms corresponding to these two *m*/*z* from LC-MS/MS (Liquid Chromatography—Tandem Mass Spectrometry) analyses of a plasma sample from KE-gavaged mice are illustrated in Figure 1e,f. Interestingly, redundant peaks were detected at the same retention time as KE (10.48 min) in these extracted ion chromatograms. These observations suggested that butanediol and β-hydroxybutyrate could be produced in the electrospray ionization source from KE, thus confirming the chemical composition of the ketone ester (Figure 1e,f). Relative quantification by LC-MS of the degradation products of KE showed a significant increase in the levels of butanediol (57-fold) (Figure 1g) and β-hydroxybutyrate (17-fold) (Figure 1h) in plasma samples from mice that had ingested KE compared to samples from control mice.

**Figure 2:**
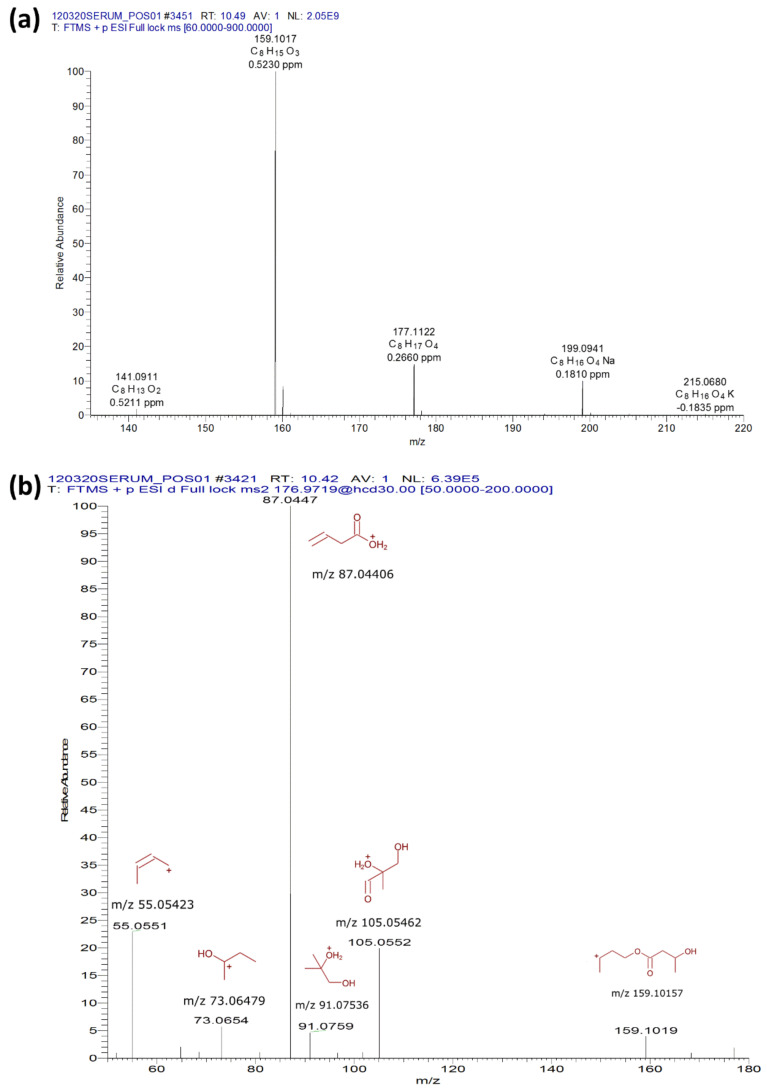
LC-MS identification of (R)-β-hydroxybutyrate and (R)-1,3-butanediol. The LC-MS identification of the KE was validated by the presence of adducts ([M+H−H_2_O]^+^, [M+H−2H_2_O]^+^, [M+Na]^+^ and [M+K]^+^) (**a**) and a MS/MS fragmentation modeled in Mass Frontier 8.0 (Slovakia) (**b**).

LC-MS identification of the KE was validated by the presence of the various adducts ([M+H−H_2_O]^+^, [M+H−2H_2_O]^+^, [M+Na]^+^ and [M+K]^+^) (Figure 2a) and MS/MS fragmentation modeled in Mass Frontier 8.0 (Slovakia) (Figure 2b).

### 2.2. Plasma Pharmacokinetics of β-Hydroxybutyrate in Non-Fasted Mice after KE Ingestion

**Figure 3:**
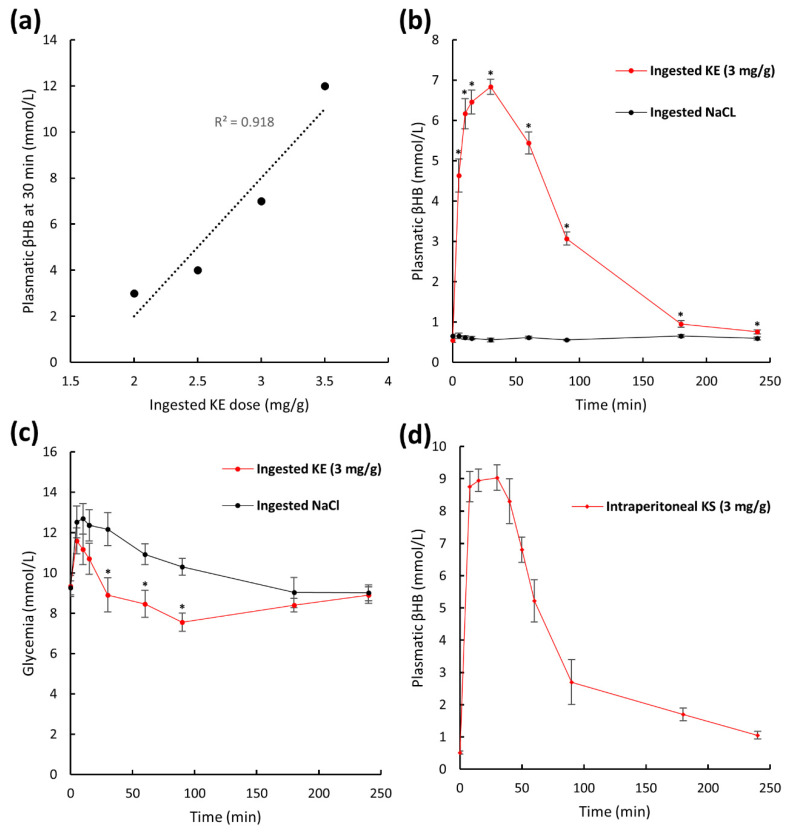
Plasma pharmacokinetics of β-hydroxybutyrate and glucose after KE ingestion. Plasma levels of βHB 30 min after intake of 2, 2.5, 3, and 3.5 mg ketone ester (KE)/g of body weight (**a**). Plasma βHB pharmacokinetics after intake of 3 mg KE/g of body weight or 0.9% NaCl (**b**) and glycemia with the same animals (n = 10) (**c**). Plasma βHB pharmacokinetics of mice after intraperitoneal injection of ketone sodium salt (3 mg KS/g of body weight) (*n* = 10) (**d**). * *p* < 0.05.

We also studied the plasma pharmacokinetics of β-hydroxybutyrate in mice following KE ingestion. For humans, plasma β-hydroxybutyrate levels range from 0.2 to 0.5 mmol/L but levels can reach 5 to 7 mmol/L during fasting. A level of 5.5 mmol/L can also be achieved by repeated KE ingestion (three daily doses of 714 mg/kg of body weight for 5 days) which has been described as well tolerated [15]. Ketone levels from 10 to 20 mmol/L are observed in subjects suffering from diabetes and, they are considered pathological. We therefore aimed at achieving a βHB plasma level of at least 5 mmol/L and not exceeding 8 mmol/L to avoid possible toxic effects due to metabolic acidosis. We first determined the dose of ingested KE needed to obtain ketosis of 5 to 8 mmol βHB/L in mice. As described in the literature, the maximal KE concentration in the plasma commonly occurs 30 min after KE ingestion [15]. We therefore measured plasma βHB levels in blood obtained from the mouse tail vein by an enzymatic method 30 min after ingestion of 2, 2.5, 3, and 3.5 mg KE/g of body weight. We found a linear correlation (R^2^ = 0.918) between the dose of ingested KE and the βHB concentration in the plasma (Figure 3a). At an oral gavage KE dose of 3 mg KE/g of body weight, a ketosis similar to that achieved in humans during fasting or by repeated KE intake of 6.83 ± 0.19 mmol βHB/L was obtained 30 min after KE ingestion and was thus chosen for our pharmacokinetic and pharmacodynamic studies. Plasma βHB pharmacokinetics of KE-gavaged (3 mg KE/g of body weight) and NaCl-gavaged control mice is shown in Figure 3b. Before KE ingestion (t = 0 min) βHB plasma levels were low (0.54 ± 0.04 mmol/L). As mentioned above, the maximum (Cmax) of 6.83 ± 0.19 mmol/L βHB was obtained 30 min (Tmax) after KE gavage and remained above 1 mmol/L for up to 180 min. In control mice, plasma βHB levels did not change significantly after NaCl ingestion (Figure 3b). Because the effects of exogenous ketones on glycemic regulation have been reported by other groups [19,20,21], we also measured the animals’ plasma glucose levels (Figure 3c). At the Tmax of βHB (t = 30 min), and for a subsequent 90 min time period, we found that the plasma glucose levels were significantly lower in the KE-gavaged mice compared to the control mice (at Tmax = 30 min: 8.91 ± 0.84 and 12.17 ± 0.81 mmol/L, respectively, *p* = 0.009; Figure 3c).

We also established the pharmacokinetic of intraperitoneally administered ketone sodium salt solution (KS) commonly used in ischemic neuroprotective studies [6]. The animals received a single dose of 3 mg KS/g of body weight. The obtained kinetic profile (Figure 3d) was similar to the kinetic profile obtained with KE-gavaged mice (Figure 3b) and, the maximum plasma ketosis was reached at 30 min (Tmax) after the ketone administration for both groups. The injected KS was a racemic mixture of the two optical isoforms of βHB, d-βHB, and l-βHB, whereas the oral KE was a non-racemic solution of (R)-3-hydroxybutyl (R)-3-hydroxybutyrate. The enzyme-based assessment of βHB did not allow to discriminate the two βHB isoforms. Thus, we cannot quantitively compare the plasma βHB levels of KS-injected mice with those of KE-gavaged mice. Nevertheless, the results showed that ketosis could be obtained by an easy oral intake of ketones in their esterized form (up to 6.84 ± 0.19 mmol/L) equally as rapidly as by the more intrusive injection of ketone sodium salts.

### 2.3. Brain β-Hydroxybutyrate Levels in KE-Gavaged Mice

**Figure 4:**
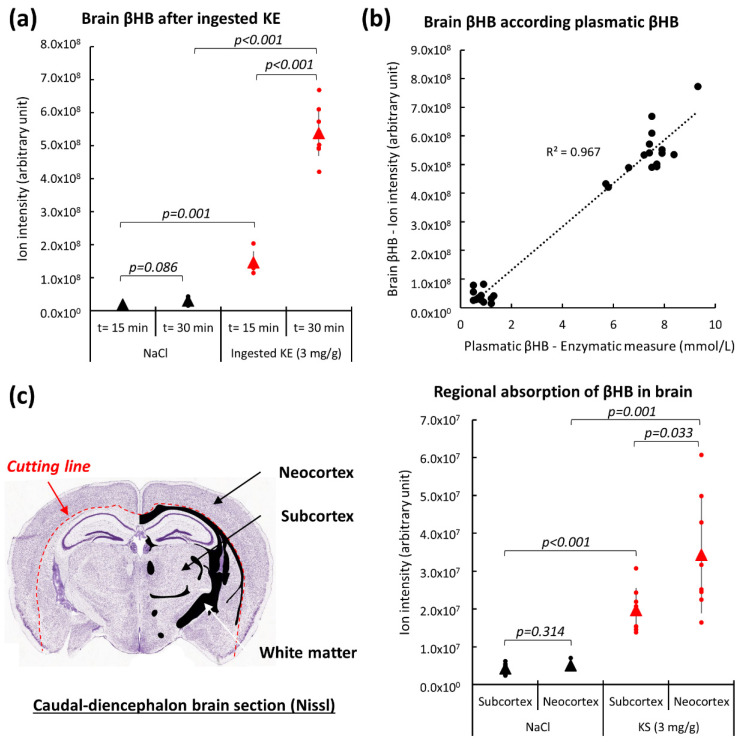
Brain βHB levels after KE ingestion. LC-MS analyses of brain βHB levels at 15 min and 30 min after intake of 3 mg KE/g of body weight and 0.9% NaCl (n = 10) (**a**). Brain βHB levels at different plasma βHB concentrations (n = 10) (**b**). Regional brain βHB levels 15 min after IP injection of 3 mg ketone salt/g of body weight (n = 8). LC-MS analyses were conducted on frozen sections after separation (red dotted line) of neocortex (grey matter) from the subcortex (grey and white matter) region using microdissection as specified on the cutting plane above (**c**).

We subsequently studied βHB pharmacokinetics after oral KE-gavage in the brains of non-fasted mice. βHB levels were assessed by LC-MS on homogenized whole mouse brains from animals at 15 min and 30 min (Tmax) after KE-ingestion (3 mg KE/g of body weight) and NaCl-ingestion (0.9%). As shown in Figure 4a, the βHB levels in the brains from mice 15 min after KE-ingestion was 2.3-fold higher than in the brains from control animals. The levels then increased rapidly to 18-fold above those obtained for the control animals at Tmax (t = 30 min). In addition, at t = 30 min after KE gavage we found a linear correlation between brain βHB levels (assessed by LC-MS) and plasma βHB levels (assessed by enzymatic assay) (Figure 4b). We concluded that the passage of βHB into the brain largely depended on the amount of βHB present in the blood and, thus assumed that βHB easily crossed the blood-brain barrier. Similar results were observed for butanediol (Supplementary Figure S1a). The accumulation of butanediol in the brains of animals after KE-ingestion is consistent with our previous results obtained with the plasma of these animals. The plasma butanediol levels in KE-gavaged mice increased since butanediol was not completely metabolized into βHB in the liver or in the blood and was able to subsequently cross the blood-brain barrier.

We also studied the regional absorption of βHB in the mouse brain. βHB levels were assessed by LC-MS from caudo-diencephalon frozen sections after separation of the neocortex from the subcortical region using micro-dissection of frozen brain slices as specified in Figure 4c. Significant higher levels of *N*-acetyl-aspartate (NAA, a neuronal biomarker [22]) (Supplementary Figure S1b) were found in samples obtained from the neocortex when compared to the subcortical regions. This suggested microdissection of the frozen brain slices enrichening gray matter in neocortex samples and white fiber tracts in subcortex samples. Interestingly, in the neocortical regions from KE-gavaged mice, βHB levels were significantly higher than in the subcortical regions (Figure 4c). No significant difference in the βHB levels of these regions was found in the brains of control animals (Figure 4c).

### 2.4. Energy Metabolism in the Brain of KE-Gavaged Mice

**Figure 5:**
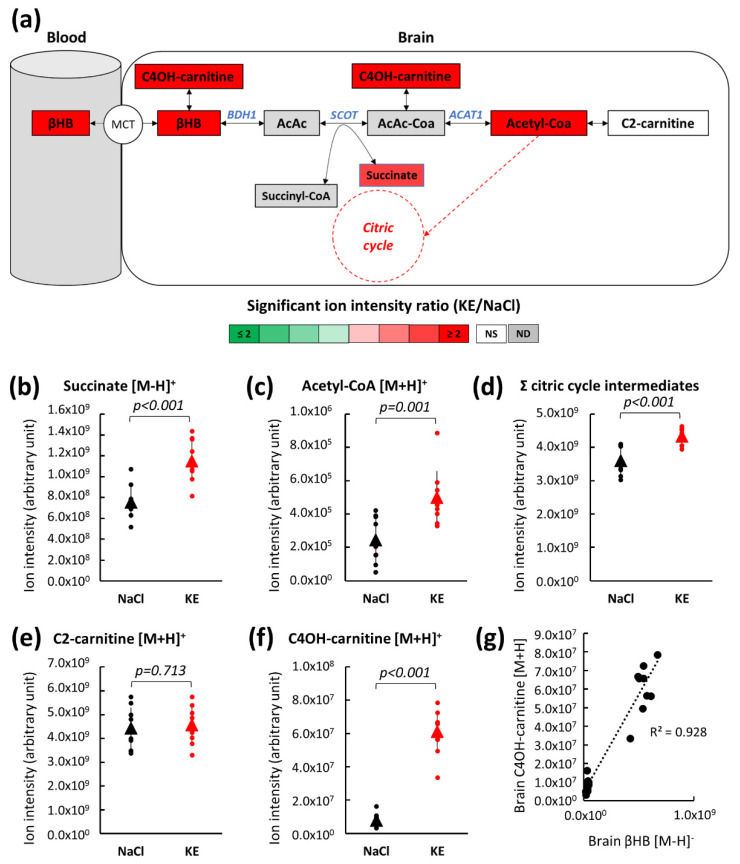
Brain metabolite levels of the βHB pathway after KE ingestion. Schematic representation of the brain βHB pathway indicating significant metabolic changes for the relevant intermediates by color code. Brain metabolite levels were assessed by LC-MS 30 min after the ingestion of KE (3 mg KE/g of body weight) or 0.9% NaCl (**a**). Individual LC-MS analysis of succinate (**b**), acetyl-CoA (**c**), total citric acid cycle intermediates (**d**), C2-carnitine (**e**), and C4OH-carnitine (**f**). Correlation between βHB and C4OH-carnitine in brain 30 min after the ingestion of KE (n = 10) (**g**). (ND: not detected; NS: not significant; MCT: monocarboxylate transporters; C4OH-carnitine: hydroxybutyrylcarnitine; AcAc: acetoacetate; AcAc-CoA: acetoacetyl-CoA; C2-carnitine: acetyl-carnitine; BDH1: β-hydroxybutyrate dehydrogenase 1; SCOT: succinyl-CoA:3-ketoacid CoA transferase; ACAT1: acetyl-CoA acetyltransferase or acetyl-CoA thiolase).

Ketone body oxidation occurs in various tissues including the brain as a means of energy transformation. The mitochondrial βHB degradation pathway leads to the production of acetyl-CoA, an energy crossroad metabolite (Figure 5a). First, β-hydroxybutyrate dehydrogenase (BDH1) converts β-hydroxybutyrate into acetoacetate (AcAc). In a second step, succinyl-CoA:3-ketoacid-coenzyme A transferase (SCOT) uses succinyl CoA (an intermediate of the citric acid cycle) to convert acetoacetate into acetoacetyl-CoA (AcAc-CoA) and succinate (which is also a citric acid cycle intermediate). Finally, acetoacetyl-CoA acetyltransferase also known as acetyl-CoA thiolase (ACAT1) hydrolyzes acetoacetyl-CoA into two molecules of acetyl-CoA. Acetyl-CoA then enters the citric acid cycle, ultimately leading to ATP production, or it is converted to its storage form, acetyl-carnitine (C2-carnitine). The levels of intermediary metabolites assessed by LC-MS in whole mouse brains collected 30 min (Tmax) after oral KE (3 mg KE/g of body weight) or NaCl gavage is shown in Figure 5b–f.

KE ingestion led to an increase in the βHB level in the brain and also to a significant increase in acetyl-CoA (2.04-fold, *p* = 0.001; Figure 5c) and the citric acid cycle intermediate, succinate (1.53-fold, *p* < 0.001; Figure 5b) levels. The total amount of the citric acid cycle intermediates (Figure 5d) (for details see Supplementary Figure S2) was significantly higher (+20%) in KE-gavaged mice than in control mice. We did not detect an increased level of C2-carnitine. These findings suggested that, in non-fasted mice, acetyl-CoA produced from the increased amounts of βHB was not converted to its storage form, C2-carnitine, but entered the citric acid cycle and thus, fueled the energy metabolism.

The level of hydroxybutyrylcarnitine (C4OH-carnitine), the acylcarnitine form of βHB, was higher in brains from KE-gavaged mice than from control mice (Figure 5f). C4OH-carnitine is formed from βHB by carnitine acyltransferase in a reversible enzymatic reaction. We found a linear correlation (R^2^ = 0.928) between the level of βHB and its acylcarnitine form suggesting that excess βHB was converted to its storage form, i.e., C4OH-carnitine in the brain of KE-gavaged mice (Figure 5g).

**Figure 6:**
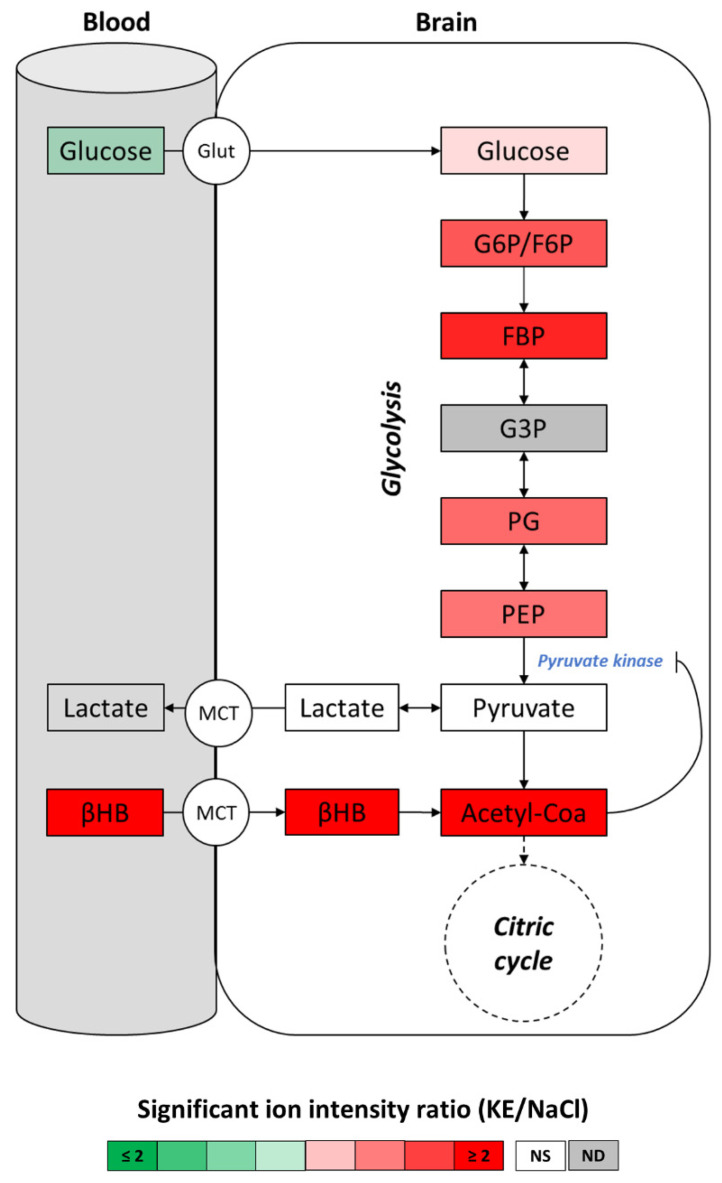
Brain metabolite levels of the glycolytic pathway after KE ingestion. Levels of brain glycolytic intermediates were assessed by LC-MS 30 min after the ingestion of KE (3 mg KE/g of body weight) or 0.9% NaCl. Plasma glucose and βHB levels were measured using an enzymatic assay shortly before cerebral dislocation (*n* = 10). ND: not detected; NS: not significant. (MCT: monocarboxylate transporters; Glut: glucose transporters; G6P: glucose-6-phosphate; F6P: fructose-6-phosphate; FBP: fructose-1,6-biphosphate; G3P: glyceraldehyde-3-phosphate; PG: phosphoglycerate; PEP: phosphoenolpyruvate).

In general, glucose is the main fuel in the brain. However, several studies show that under certain physiological conditions when glucose utilization is impaired ketones could function as the brain’s main alternative energy source to glucose [23,24,25]. In our study, animals had free access to food and did not suffer glucose deficit in the brain before or after ketone administration. Glucose degradation starts through glycolysis and acetyl-CoA is produced through the oxidative decarboxylation of pyruvate (by pyruvate dehydrogenase complex), the end-product of glycolysis. We assessed the metabolites of the glucose degradation pathway by LC-MS to determine if the high level of acetyl-CoA in the brain of KE-gavaged mice was due to increased glycolytic pyruvate production. Our results (Figure 6; details are given in Supplementary Figure S3) showed that, in the brains from these animals, pyruvate and lactate levels were not significantly different from the pyruvate and lactate levels in the brains from the control mice. This finding further strengthened our hypothesis that the high levels of acetyl-CoA in the brains from KE-gavaged mice compared to control animals was based on the degradation of the higher amount of βHB, rather than increased glycolytic flux and pyruvate production. While KE gavage had no effect on the pyruvate level, we observed an accumulation of glycolytic intermediates upstream of pyruvate. These results suggested an inhibition of pyruvate kinase activity via negative feedback caused by high levels of acetyl-CoA as described by Valente-Silva P. et al. and Weber G et al. [26,27]. Interestingly, we previously found that oral KE gavage led to a decrease of the plasma glucose level (Figure 3c) at Tmax, whereas the glucose level in the brain was simultaneously slightly increased. This finding was consistent with the observed reduction of the glycolytic flux, suggesting glucose sparing in the brain after KE ingestion.

## 3. Discussion

Here, we report the rapid increase of the acetyl-CoA levels in the mouse brain after a single oral intake of the ketone ester (R)-3-hydroxybutyl (R)-3-hydroxybutyrate. We obtained strong evidence that the increased level of acetyl-CoA in the brain fueled the citric acid cycle and allowed glucose sparing through negative feedback on the glycolytic flux. The resulting neuroprotective effect of the ketone ester makes it a promising agent for the reduction of non-reversible brain damage.

Ketone body metabolism is a survival trait conserved in higher organisms to prolong life during an energy challenge or a metabolic crisis [1]. In response to low glucose availability such as fasting, starvation, or untreated diabetes mellitus, ketone bodies produced by the liver mostly from fatty acids act mainly as cellular respiratory fuels to power oxidative phosphorylation. The main benefit of ketone bodies is their ability to provide an alternative energy source to glucose for mitochondrial ATP production in extrahepatic tissues like brain, heart, or skeletal muscle [1]. Under normal physiological conditions, the constant production of ketone bodies by the liver on the one hand and their constant utilization by extrahepatic tissues, on the other hand, leads to a low level of ketone bodies in the blood (<1 mmol/L). Ketosis, i.e., a significant increase of the circulating plasma ketone bodies occurs in response to long-lasting carbohydrate deprivation or an energy deficit that induces increased hepatic ketone production.

In addition to their role as energy metabolites, cellular signaling functions have been reported for ketone bodies [5]. More recently, the beneficial effects of ketosis for human health and particularly in the treatment of neurological diseases have become a subject of growing interest. In the past, ketosis was commonly induced by carbohydrate deprivation, referred to as nutritional ketosis. This ketogenic diet generates mild ketosis (1–3 mmol/L) about 72 h after the onset of nutritional carbohydrate withdrawal and is therefore not a suitable treatment in the case of acute neurological diseases. To accelerate the achievement of ketosis and to avoid the inconvenience of adhering to dietary restrictions, the administration of exogenous ketones was proposed. These exogenous ketones are either ketone bodies (principally βHB) or βHB precursors such as ketone mineral salts in oral or injectable forms. More recently, Clarke et al. generated an oral form of a ketone monoester ((R)-β-hydroxybutyl (R)-3-hydroxybutyrate) by trans-esterifying ethyl (R)-β-hydroxybutyrate with the ketone precursor (R)-1,3-butanediol [15]. To date, published data have proven this ketone ester’s safety and tolerability for humans [28,29,30]. The agent is thus considered to be a safe food supplement (GRAS: Generally Recognized as Safe) by the U.S. Food and Drug Administration (FDA).

In this study, we first analyzed the plasma pharmacokinetics and pharmacodynamics of the ketone ester. We showed by LC-MS analysis the appearance of βHB and the precursor butanediol in the plasma of animals oral-gavaged with (R)-β-hydroxybutyl (R)-3-hydroxybutyrate ketone monoester. We assume that the latter was hydrolyzed into βHB, and the βHB precursor butanediol, most probably in the gut and the blood by non-specific esterases [16]. Similar to results obtained in humans, we found that the ingestion of this ketone ester induced a rapid and significant ketosis in mice [15]. Within 30 min (Tmax) after the KE intake (3 mg KE/g of body weight), the plasma levels of βHB in the treated animals reached a maximum of 6.83 ± 0.19 mmol/L, which was consistent with the levels of ketosis achieved in humans during fasting or by repeated KE intake. Ketosis was sustained above 1 mmol/L for as long as 180 min. The pharmacokinetic profile obtained for intraperitoneal administration of the ketone sodium salt (Na-βHB) was similar to that of the oral intake of the ketone monoester. In both conditions, Tmax was reached 30 min after ketone administration. Contrary to Na-βHB, KE was hydrolyzed into βHB and the βHB precursor butanediol. In contrast to findings of several groups using high doses of KE [15,17], under our experimental conditions, butanediol was not completely metabolized into βHB via first-pass metabolism in the liver of the KE-gavaged mice [17,18]. The accumulation of butanediol in the plasma and in the brain at Tmax observed in this study supported the hypothesis that the application of a KE dose of 3 mg KE/g of body weight was exceeding the metabolic capacity of the liver in mice [15].

Interestingly, at Tmax, we noted transient hypoglycemia (lasting for about 1 h) in the KE-gavaged mice that was not detected in control mice. Previous clinical or pre-clinical studies report a correlation between the acute elevation in blood ketone concentrations achieved by exogenous ketones and the reduction in blood glucose [19]. The underlying molecular mechanisms remain unclear, but some studies suggest that ketone supplementation increases insulin sensitivity or reduces hepatic glucose output [20,21]. However, Clarke et al. did not report glucose levels below 25 mmol/L in humans nor did they detect any clinical symptoms during ketosis at maximum plasma βHB levels of 5.5 mmol/L [15].

We then studied the effects of rapid ketosis of 6.83 ± 0.19 mmol βHB/L on the brain at Tmax which was induced by KE gavage of non-fasting mice. It is known that ketone bodies derived from both, endogenous and exogenous sources are equivalently metabolized into βHB, which passes into the brain by crossing the blood-brain barrier via monocarboxylate transporters (MCTs) [31]. As reported in the literature, we found that the βHB uptake into the brain positively correlated with the βHB concentration in the blood [1,32]. According to our results, plasma butanediol also crossed the blood-brain barrier where it was converted into βHB most probably by alcohol and aldehyde dehydrogenases. These enzymes function predominantly in the liver, but they are also found in the brain [17,33].

We also found that βHB was not homogenously transported into the different brain regions. βHB accumulation was significantly higher in the neocortex than in the subcortex. This finding was in accordance with results previously reported by Hawkins et al. [34]. The authors showed that the cerebral cortex has the highest capacity of ketone body transport activity of all brain regions [34,35], while uptake is low in the corpus callosum and other white-matter structures, mainly due to MCT expression [2,36,37].

In extrahepatic tissues, endogenous or exogenous βHB is converted into two acetyl-CoA molecules which can enter the citric acid cycle and be used for fatty acid synthesis or, alternatively, enter the mevalonate pathway leading to the synthesis of cholesterol [1,3,38]. In this study, we assessed the levels of metabolites in the brain of KE-gavaged mice. Cerebral energy metabolism was assessed by untargeted metabolomic LC-MS analyses with whole brains collected 30 min (Tmax) after oral KE gavage of mice given free access to food and drinking water and not exposed to any dietary restrictions. We found, that under these conditions, exogenous βHB led to an increase in the cerebral acetyl-CoA and the citric cycle intermediate levels and of the hydroxybutyrylcarnitine level. We thus assumed that the βHB was partly converted into acetyl-CoA which fed the citric cycle and partly into its storage form, hydroxybutyrylcarnitine. Certain physiological conditions like fasting or ketogenic diets lead to an increase in the plasma or muscle C2-carnitine levels. However, in the brains of our non-fasting KE-gavaged mice ketosis led to an increased acetyl-CoA level but interestingly, the acetyl-CoA was not stored as its acetyl form, C2-carnitine, but entered the citric cycle. In the brain, hydroxybutyrylcarnitine is synthesized via a reversible enzymatic reaction when an excess of exogenous βHB needs to be buffered [39]. Under standard physiological conditions, acetyl-CoA is mainly produced by pyruvate dehydrogenase complex (PDC) from pyruvate, the end product of glycolysis. Because the level of pyruvate (and lactate) in the brain of the KE-gavaged mice was not significantly different from that of control mice, we concluded that the increased level of acetyl-CoA in the brain of the KE-gavaged animals originated from the intake of exogenous βHB. We also observed an accumulation of glycolytic intermediates upstream of pyruvate. These results suggested a feedback inhibition of pyruvate kinase, which converted phosphoenolpyruvate to pyruvate, which then led to acetyl-CoA as described previously [26,27]. Moreover, we observed an upward trend in the glucose levels in the brains of KE-gavaged mice, suggesting glucose sparing due to the reduction of the glycolytic flux. Brain ketone bodies are an alternative energy source to glucose under certain physiological conditions such as carbohydrate deprivation or when glucose utilization is impaired [23,24,25]. Here, we show that exogenous βHB functioned as a cerebral fuel under normal physiological conditions, i.e., when glucose is available. Moreover, we obtained evidence that the exogenous βHB was the preferred carbon source in the brain of the KE-gavaged, non-fasted mice. Our findings strengthen the hypothesis of Valente-Silva P. et al. [26] proposing that ketone bodies compete with glucose for neuronal acetyl-CoA generation in rat hippocampal slices perfused with exogenous βHB. So far, it had not been possible to deduce from previous in vivo studies the extent to which the exogenous βHB may feed the citric acid cycle and competes with glucose. Our study provides important new pre-clinical data on the effects of exogenous ketones on cerebral energy metabolism and gives new insights into the underlying molecular mechanisms of their beneficial role in neuroprotection.

Today, the way in which ketone bodies contribute to cerebral energy homeostasis is of ever-growing interest, particularly in aging and neurodegenerative diseases because mitochondrial dysfunction and altered glucose metabolism are thought to play important roles in the progression of the aging process and of various chronic neurological diseases [3,4,5,6,7]. Likewise, more and more studies have addressed the potential role of ketone bodies in neuroprotection during acute neurological diseases [5,6,7]. Since the 1980s, several pre-clinical studies have consistently shown the potential for ketogenic therapy in the acute neuroprotection of cerebral ischemic injuries in animal models [8,9,10,11,12,13]. In a systematic review, White et al. describe the acute neuroprotective effect of ketogenic therapy based on the reduction of the ischemic lesion volume and better functional outcomes [5]. To our knowledge, the translation of these results into clinical studies was not proposed due to the ketosis induction methods used in these animal models that are not suitable for use with humans. Applying a ketogenic diet is not suitable in the case of acute diseases because of the delayed onset of ketosis. Rapid and significant ketosis could be obtained by intraperitoneal injection of ketone salts, but this is not considered a safe procedure for humans in a pathological condition. Alternatively, ketone esters generated recently by Clarke et al. [15] and considered to be a safe food supplement by the U.S. Food and Drug Administration allow for rapid and significant ketosis in a single oral intake and thus, might be considered as promising neuroprotective agents in acute stroke. Ischemic stroke is caused by a reduction in the blood flow to the brain leading to an energy crisis due to a disruption in the balance of energy demand and supply. Hyperglycemia in acute stroke is considered to be a robust predictive factor of bad clinical outcome [40,41]. In this situation, the glucose level in the brain is high while the oxygen level is low, leading to an increased lactate production by anaerobic glycolysis and the consequent acidification of ischemic tissue. For this reason, hyperglycemia in acute stroke causes the rapid growth of ischemic injuries [42,43]. We here report strong evidence that exogenous βHB competes with glucose for acetyl-CoA generation in the mouse brain. We hypothesize that this pharmacodynamic property of exogenous ketone bodies explains, in part, the reduction of stroke volume during ketogenic therapy reported in previous preclinical studies [5,8,13]. Our results define the rationale of the second part of our KETOSTROKE project, to investigate the potential role of ketone esters in neuroprotection using a mouse MCAO (middle cerebral artery occlusion) model of acute stroke.

## 4. Materials and Methods

### 4.1. Animals

This study comprises the first part of our KETOSTROKE project in SLC5A8-deficient transgenic mice, a model with permanent ketone body deficiency [44]. We therefore used wild-type animals with the same genetic background as our SLC5A8-deficient animals for all experiments in this study. For the generation of this strain, see Suissa L. et al. (2019) [44]. The SLC5A8-deficient animals initially generated in a mixed C57BL6/129Sv background were back-crossed with commercial C57BL6/J (Janvier laboratories, Le-Genest-Saint-Isle, France) for seven generations. Then heterogenous males and females were mated to obtain homozygous control (wild-type) and homozygous SLC5A8-deficient mice. The homozygous wild-type mice were bred to produce the male animals used for our studies. Animals were fed a standard diet and given free access to food and drinking water throughout the study.

All study protocols followed the ARRIVE guidelines (Animal Research: Reporting of In vivo Experiments) and were approved by the French Ministry of Education and Research (number 23313-2019120622578192, approval date 22 January 2020) that adheres to the politics and guidelines of the National Institute of Health principles of animal laboratory care (NIH publication 86-23, revised 1995). The animals were treated in accordance with the French Agricultural Ministry guidelines, and the experiments were approved by the University of Nice Sophia Antipolis Animal Care User and Ethics Committee (Ciepal, reference NCE/2019–638).

### 4.2. Ketone Administration

3-month-old non-fasted male mice were oral gavaged with solutions of 2.0, 2.5, 3.0, and 3.5 mg (R)-3-hydroxybutyl (R)-3-hydroxybutyrate (H.V.M.N. Ketone Ester^®^, H.V.M.N., San Francisco, CA, USA) per gram of body weight or with the same volume of 0.9% NaCl using a metal gavage needle (randomization 1:1). Alternatively, 3-month-old non-fasted male animals were injected intraperitoneally with 3 mg dl-β-hydroxybutyrate sodium salt (SIGMA) per gram of body weight in 0.9% NaCl or with the same volume of 0.9% NaCl only (randomization 1:1).

### 4.3. Blood Collection and Processing for LC-MS Analysis

Mice were sacrificed by cervical dislocation 15 min after oral gavage. Blood was collected from sacrificed animals and incubated at room temperature for 30 min. After centrifugation at 3000× *g* for 20 min et 4 °C, supernatants were removed, mixed with five volumes of methanol and incubated overnight at −20 °C for protein precipitation. Samples were then centrifuged at 13,000× *g* for 15 min at 4 °C. Supernatants were vacuum-dried, resuspended in 80 µL of a 20:80 acetonitrile-H2O mixture (HPLC grade, Merck Millipore, Darmstadt, Germany), and stored at −20 °C until use for LC-MS analysis.

### 4.4. Brain Collection and Processing for LC-MS Analysis

Mice were sacrificed by cervical dislocation 15 min or 30 min after oral gavage of 3 mg (R)-3-hydroxybutyl (R)-3-hydroxybutyrate (H.V.M.N. Ketone Ester^®^, H.V.M.N., CA, USA) per gram of body weight or with the same volume of 0.9% NaCl. For whole brain analyses, freshly collected brains were cut into two equal parts along their median line. Each part was homogenized immediately in methanol and incubated overnight at −20 °C. Samples were then centrifuged at 13,000× *g* for 15 min at 4 °C. Supernatants were removed, vacuum-dried, resuspended in 80 µL of a 20:80 acetonitrile-H2O mixture (HPLC grade, Merck Millipore), and stored at −20 °C until use in LC-MS analysis. For regional analyses, whole brains were removed 15 min after intraperitoneal injection of 3 mg DL-β-hydroxybutyrate sodium salt (SIGMA-ALDRICH, Saint-Louis, MO, USA) per gram of body weight in 0.9% NaCl or with the same volume of 0.9% NaCl and rapidly frozen in liquid nitrogen. 10 µm sections were prepared from frozen brains with a cryostat, placed on glass slides and stored at −20 °C until use. Specific parts of the slices were carefully removed from the slides by scraping with a scalpel. The remaining tissue was detached from the slides with 70% methanol and incubated overnight at −20 °C. Samples were then centrifuged at 13,000× *g* for 15 min at 4 °C. Supernatants were removed, vacuum-dried, resuspended in 80 µL of a 20:80 acetonitrile-H_2_O mixture (HPLC grade, Merck Millipore), and stored at −20 °C until use in LC-MS analysis.

### 4.5. LC-MS Analysis

Chromatographic analysis was performed with the DIONEX Ultimate 3000 HPLC system coupled to a chromatographic column (Phenomenex Synergi 4 u Hydro-RP 80A 250 × 3.0 mm) set at 40 °C and a flow rate of 0.9 mL/min. Gradients of mobile phases (mobile phase A: 0.1% formic acid in water and mobile phase B: 0.1% formic acid in acetonitrile) were performed over a total of 25 min. MS analysis was carried out on a Thermo Scientific Exactive Plus Benchtop Orbitrap mass spectrometer. The heated electrospray ionization source (HESI II) was used in positive and negative ion modes. The instrument was operated in full scan mode from *m*/*z* 67 to *m*/*z* 1000. High-resolution accurate mass (HRAM) full-scan MS and top five MS/MS spectra were collected in a data-dependent fashion at a resolving power of 70,000 and 35,000 at FWHM *m*/*z* 200, respectively. The post-treatment of data was performed using the MZmine2 version 2.53 (http://mzmine.github.io/; Boston, MA, USA) [45]. Metabolites were identified using the Human Metabolome Database version 4.0 [46]. LC-MS analyses were performed in a blinded assessment; that is, without revealing the identity of the samples to the mass spectroscopist prior to analyses.

### 4.6. Glucose and β-Hydroxybutyrate Enzymatic Measurements

Blood glucose and β-hydroxybutyrate concentrations were quantified in venous blood samples with enzyme-based reagent strips using a Glucometer and a Ketometer (OneTouch Verio, LifeScan Inc., Burnaby, BC, Canada), respectively. Blood samples were obtained by incision of the tail vein with a lance. 2 µL samples were drawn before (*t* = 0) and at 5, 10, 15, 30, 60, 90, 180, and 240 min after ketone or NaCl administration. Measurements were performed by researchers without knowing the treatment group.

### 4.7. Statistical Analyses

Statistical analyses were performed using Microsoft Excel software for all experiments. Continuous variables are presented as means with standard deviation. Parametric Student’s t-tests were used to compare different conditions after carrying out Shapiro–Wilk normality test. To determine linear correlations between two parameters, the coefficient of determination denoted R2 was assessed. A *p*-value < 0.05 was considered to be significant. No animals have been excluded from any of our experiments.

## 5. Conclusions

The orally ingested ketone monoester (R)-3-hydroxybutyl (R)-3-hydroxybutyrate led to rapid and significant ketosis in KE-gavaged non-fasting mice. Our results show, for the first time, that exogenous βHB led to an increase of acetyl-CoA and the citric cycle intermediates in the brain. In addition, we found that the increased level of acetyl-CoA inhibited glycolysis in a feedback reaction and thus, competed with glucose under physiological conditions. The effects of ketone ester administration on the mouse brain that we describe here strongly support the potential of these agents for neuroprotection studies in the case of acute neurological diseases.

## Data Availability

Data available on request due to restrictions eg privacy or ethical.

## Supplementary Materials

Supplementary Materials can be found at https://www.mdpi.com/1422-0067/22/2/524/s1. Figure S1: Brain butanediol levels after KE ingestion; Figure S2: Brain βHB after KE ingestion; Figure S3: Brain glucose after KE ingestion.

## Abbreviations

KE	Ketone ester
βHB	Β-Hydroxybutyrate
Acetyl-CoA	Acetyl-Coenzyme A
LC-MS	Liquid chromatography-Mass spectrometry
