# Supplementary material for: Ingested Ketone Ester Leads to a Rapid Rise of Acetyl-CoA and Competes with Glucose Metabolism in the Brain of Non-Fasted Mice
Source: Int J Mol Sci. 2021 Jan 7;22(2):524. doi: 10.3390/ijms22020524 (PMC7825708; doi:10.3390/ijms22020524)
Supplement: Supplementary file 1 [file ijms-22-00524-s001.pdf]

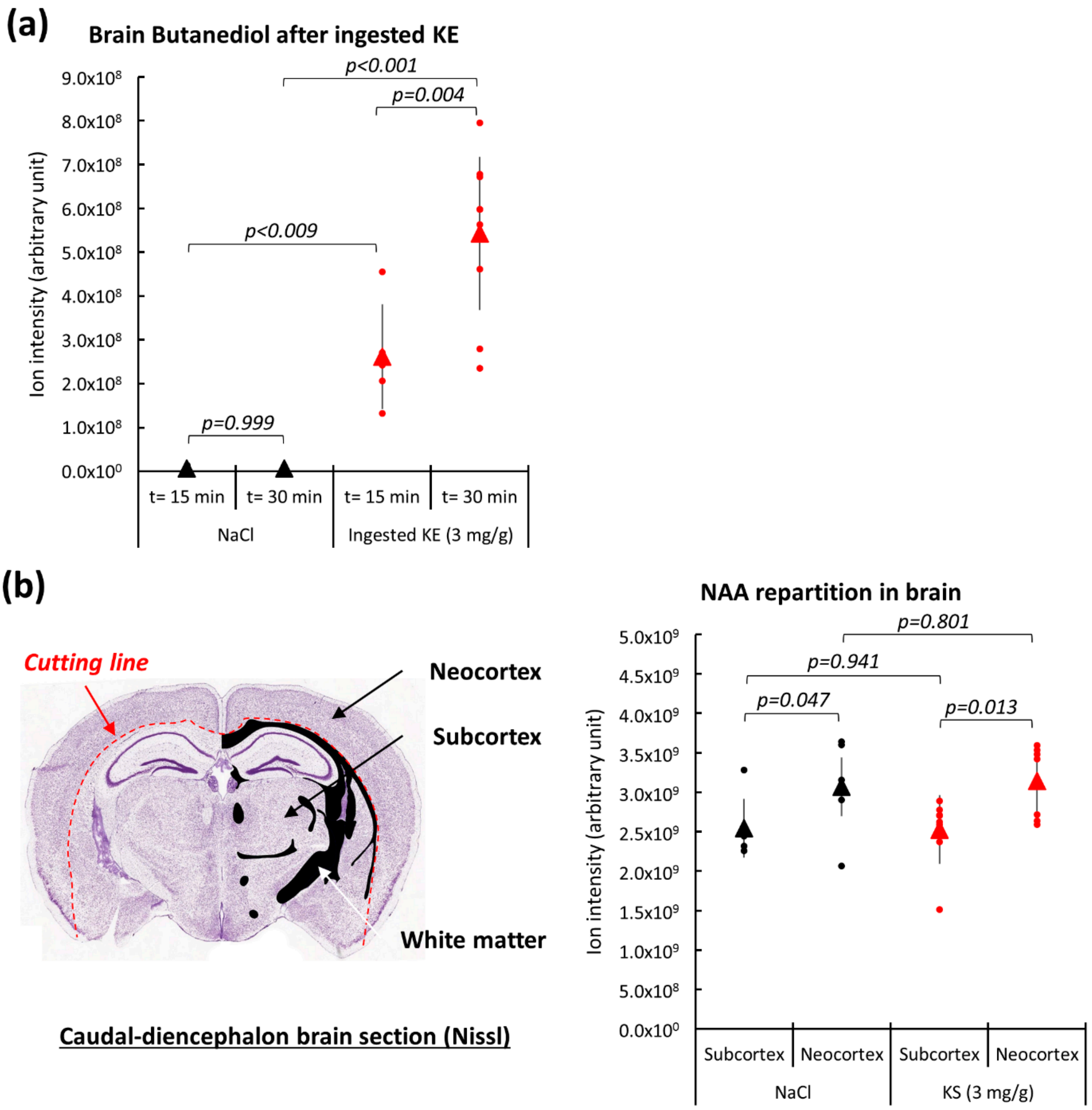

Figure S1: Brain butanediol levels after KE ingestion. Brain levels of butanediol at 15 min and 30 min after KE (3 mg/g) and NaCl intake (n=10) (**a**). Regional brain levels of NAA (Neuronal biomarker) after IP injection of 3 mg ketone salt/g at t=15 min (n=8). Measures were made by LC-MS from frozen sections after separation (red dotted line) of neocortex (grey matter) from the subcortex (grey and white matter) region using micro-dissection as specified on the cutting plane above (**b**).

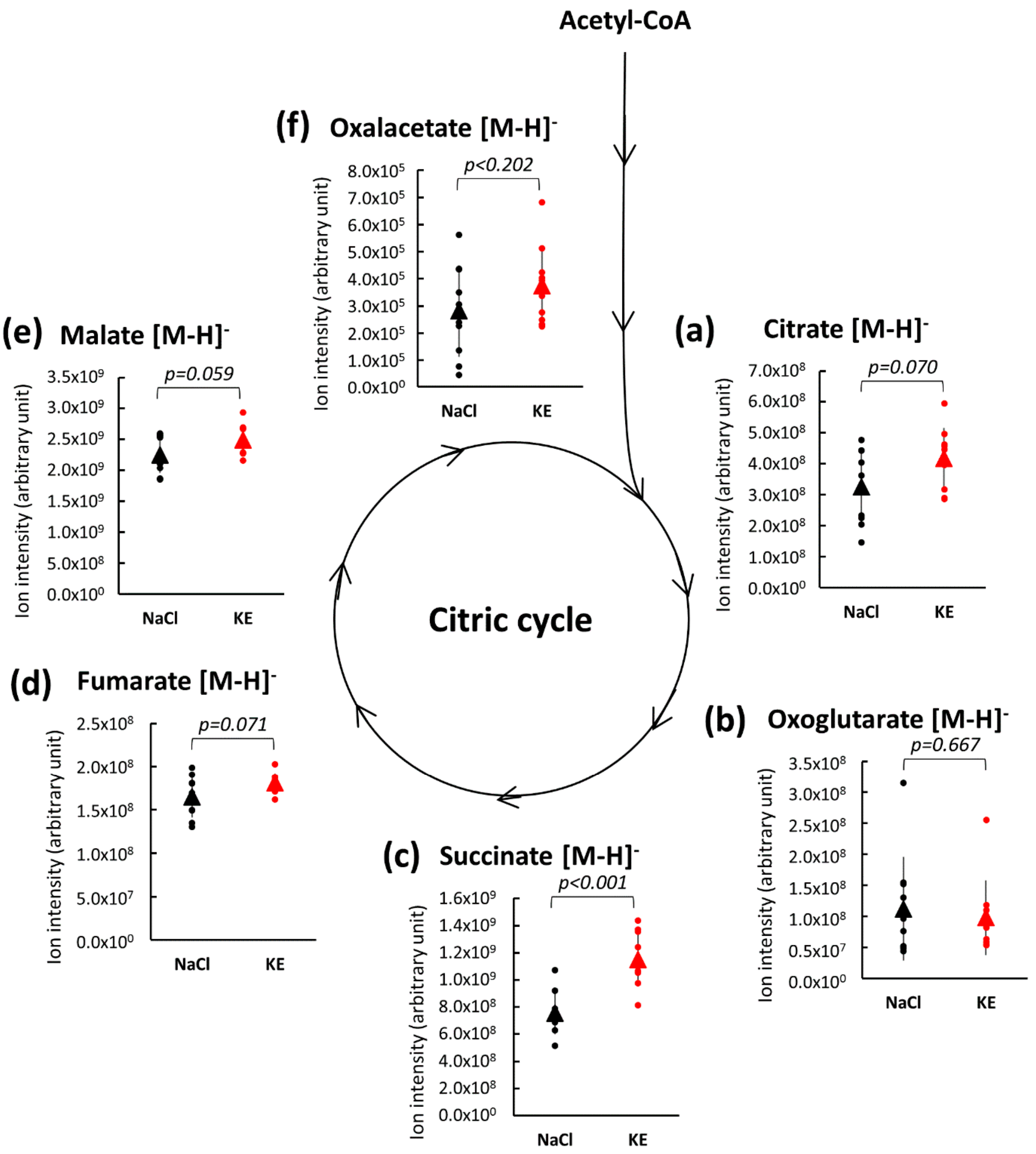

Figure S2: Brain βHB after KE ingestion. Levels of citric cycle metabolites assessed by LC-MS 30 min after the ingestion of KE (3 mg/g) or 0,9 % NaCl : citrate (**a**), oxoglutarate (**b**), succinate (**c**), fumarate (**d**), malate (**e**) and oxaloacetate (**f**).

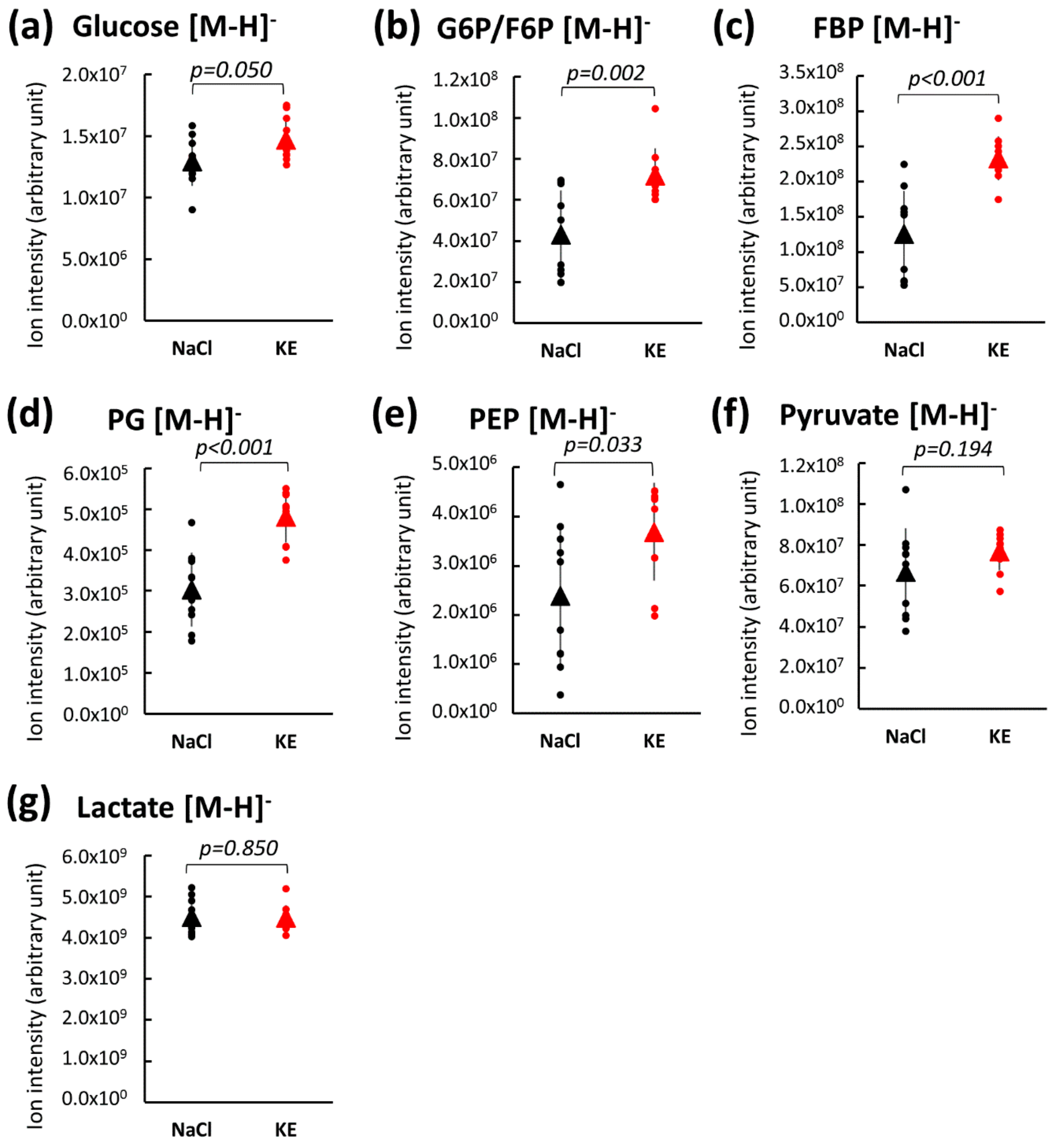

Figure S3: Brain glucose after KE ingestion. The levels of glycolytic metabolites were assessed by LC-MS 30 min after the ingestion of KE (3 mg/g) or 0,9 % NaCl (n=10): glucose (**a**), G6P/F6P (glucose-6-phosphate/fructose-6-phosphate) (**b**), FBP (fructose-1,6- biphosphate) (**c**), PG (phosphoglycerate) (**d**), PEP (phosphoenolpyruvate) (**e**), pyruvate (**f**) and lactate (**g**).
